# Haploinsufficiency of *insulin gene enhancer protein 1* (*ISL1*) is associated with d-transposition of the great arteries

**DOI:** 10.1002/mgg3.75

**Published:** 2014-04-17

**Authors:** Kazutoyo Osoegawa, Kathleen Schultz, Kenneth Yun, Nebil Mohammed, Gary M Shaw, Edward J Lammer

**Affiliations:** 1 Center for Genetics, Children's Hospital Oakland Research Institute, Children's Hospital Research Center OaklandOakland, 94609, California; 2 Department of Pathology, Stanford University School of MedicinePalo Alto, California; 3 Department of Pediatrics, Stanford University School of MedicineStanford, California

**Keywords:** Conotruncal defects, haploinsufficiency, *ISL1*, microdeletion

## Abstract

Congenital heart defects are the most common malformation, and are the foremost causes of mortality in the first year of life. Among congenital heart defects, conotruncal defects represent about 20% and are severe malformations with significant morbidity. *Insulin gene enhancer protein 1* (*ISL1*) has been considered a candidate gene for conotruncal heart defects based on its embryonic expression pattern and heart defects induced in *Isl1* knockout mice. Nevertheless no mutation of *ISL1* has been reported from any human subject with a heart defect. From a population base of 974,579 births during 1999–2004, we used multiplex ligation-dependent probe amplification to screen for microdeletions/duplications of *ISL1* among 389 infants with tetralogy of Fallot or d-transposition of the great arteries (d-TGA). We also sequenced all exons of *ISL1*. We identified a novel 20-kb microdeletion encompassing the entire coding region of *ISL1*, but not including either flanking gene, from an infant with d-TGA. We confirmed that the deletion was caused by nonhomologous end joining mechanism. Sequencing of exons of *ISL1* did not reveal any subject with a novel nonsynonymous mutation. This is the first report of an *ISL1* mutation of a child with a congenital heart defect.

## Introduction

Congenital heart defects are one of the most common birth defects (Hoffman and Kaplan 2002). Excluding infectious diseases, congenital heart defects are the leading cause of infant mortality (Malcoe et al. 1999). Approximately 20% of congenital heart defects are subgrouped as conotruncal defects, which pathogenetically result from abnormal contributions of second heart field cells and cranial neural crest cells to heart formation. Conotruncal heart defects include: tetralogy of Fallot (TOF), d-transposition of the great arteries (d-TGA), truncus arteriosus, double outlet right ventricle, and subaortic conoventricular septal defects (Ferencz et al. 1985; O'Malley et al. 1996). Of these, TOF and d-TGA comprise ∼75% of conotruncal defects.

Congenital heart defects, including conotruncal defects, have been etiologically associated with environmental, genetic factors or a combination of both (Jenkins et al. 2007). Candidate genes associated with conotruncal heart defects have been identified through various gene-hunting efforts by human genetics approaches or using model organisms. In some cases, genes were listed as candidates based on evidence from experimental model organisms, such as expression assays or gene knockouts. Among candidate genes for conotruncal heart defects that have been proposed based on murine investigations, but not yet subject to human research, we chose to investigate *ISL1* (OMIM 600366).

*ISL1* is a LIM homeodomain-containing transcription factor that was first identified as a regulator of the insulin 1 gene (Tanizawa et al. 1994). *ISL1* has been used as a marker for cardiac progenitors of the second heart field (Moretti et al. 2006; Cai et al. 2003). Mouse *Isl1*^−/−^ embryos form primitive heart tubes, but they fail to extend or loop and their right ventricles and outflow tracts are hypoplastic, or nearly absent. The heart malformations of *lsl1* knockout mice are essentially conotruncal defects (Cai et al. 2003). *ISL1* appears to be at the top of the transcriptional hierarchy that determines which splanchnic mesoderm cells will commit to cardiomyocyte differentiation and comprise the second heart field (Cai et al. 2003). It has been shown that *ISL1* binds to a 7-bp highly conserved DNA sequence on exon1 of *FGF10* and regulates *FGF10* expression during human cardiac outflow formation (Golzio et al. 2012). *ISL1* expression is, therefore, considered to be essential for generating multipotent cardiovascular progenitors within the second heart field to proliferate and migrate into the forming heart.

We, and others, have shown that array comparative genomic hybridization (array-CGH) can be successfully applied to detect chromosomal microdeletions/duplications among subjects with nonsyndromic malformations, such as cleft lip and palate and conotruncal heart defects (Osoegawa et al. 2014, 2008; Vissers et al. 2004). While genome-wide screening for microdeletions/duplications with array-CGH is a powerful investigative tool, there is a fundamental dilemma about cost versus sensitivity. Using commercially available microarrays, it is theoretically possible to detect microdeletions as small as a few kilobases, but it is financially prohibitive to screen a large population. A contrasting approach that screens candidate genes for small microdeletions/duplications complements the genome-wide approach of array-CGH. Multiplex ligation-dependent probe amplification (MLPA) was developed to identify copy number variation of targeted DNA sequences such as candidate genes (Koolen et al. 2004; Bunyan et al. 2004; Schouten et al. 2002; Zhi 2010). MLPA has evolved into a popular method to identify microdeletions/duplications from known candidate genes in a clinical and research environment (Koolen et al. 2004).

We screened DNA samples from 389 California subjects who were born with TOF or d-TGA using MLPA to detect exonic microdeletions/duplications of *ISL1*. We also sequenced exons of *ISL1* in each case to search for nucleotide changes that may result in changing the functionality of ISL1. We found a novel 20-kb deletion of *ISL1* from a subject with d-TGA.

## Material and Methods

### Study population

Research protocols for this study were approved by the California Committee for the Protection of Human Subjects as well as the Institutional Review Boards at Children's Hospital and Research Center Oakland and at Stanford University. We used data derived from a population-based case–control study, California Study of Birth Defects Causes, as described elsewhere (Lammer et al. 2009; Shaw et al. 2010). Briefly, the study population was defined by pregnant women who lived in Los Angeles, San Francisco, or Santa Clara counties, and who delivered between July 1999 and June 2004. The population included 968,885 live-born infants and 5694 stillborn fetuses. Infants and fetuses born with conotruncal heart defects were ascertained by active surveillance by staff of California Birth Defects Monitoring Program (CBDMP). We successfully matched and retrieved newborn bloodspots for 225 (89%) subjects with TOF and 164 (85%) with d-TGA totaling 389 study subjects. No parental DNA was available.

### DNA isolation and whole genome amplification

DNA was extracted from archived Guthrie cards obtained from the 389 subjects. Two 3-mm punches from each card were incubated with the GenSolve reagent (IntegenX, Pleasanton, CA) for 1 h in a shaking heat block at 65°C. After centrifugation to remove the paper, elute was purified using the QIAamp DNA Mini Kit (Cat. No. 51306; Qiagen, Germantown, MD) according to the manufacturer's instructions, with the modification that the elution step was performed with preheated buffer at 70°C, followed by incubation of the spin columns at 70°C for 10 min. DNA was recovered by centrifugation. DNA concentrations were measured using the PicoGreen method (Life Technologies, Carlsbad, CA).

Genomic DNA was directly used for MLPA experiments. Whole genome amplified (WGA) DNA was used for array-CGH, Single-nucleotide polymorphism (SNP) genotyping, PCR breakpoint junction cloning experiments, and sequencing. Genomic DNA was amplified using GenomePlex kit (Sigma, Saint Louis, MO) and purified using Sephadex G50 spin column for array-CGH and PCR breakpoint junction cloning experiments. Similarly, we amplified genomic DNA using REPLI-g Mini Kit (QIAGEN) for SNP genotyping and DNA sequencing.

### Multiplex ligation-dependent probe amplification

Reference DNA sequences containing *ISL1* (NM_002202.2) were downloaded from the University of California Santa Cruz (UCSC) Genome Browser. SNPs or repetitive sequences were masked, and the remaining nonmasked sequences were used to design the synthetic MLPA primers. Three of the six *ISL1* exons were suitable as targets for MLPA probe annealing sites. We limited the length of the probe to be <150 bases. The MLPA primers were designed using the Human MLPA Probe Design software from the Genomics Core Facility at Stony Brook University (http://genomics01.arcan.stonybrook.edu/mlpa2/cgi-bin/mlpa.cgi, Zhi 2010). Default settings were used, and no stuffer sequences were employed. When the oligonucleotides were synthesized, universal primer annealing sites GGGTTCCCTAAGGGTTGGA and TCTAGATTGGATCTTGCTGGCAC were attached to the 5′-end of the left probe and to the 3′-end of the right probe, respectively. The ligated products were designed to differ by four bases to obtain distinct separation between the peaks during capillary electrophoresis. We designed four probes for three exons of *ISL1* with lengths of 96, 116, 132, and 136 nucleotides (Table 1). We also included two probes from *T-box 1* (*TBX1*) at the length of 108 and 120 nucleotides. These *TBX1* probes were used as positive controls to monitor the performance of MLPA from a subject who had a typical 3-Mb chromosome 22q11.21 microdeletion (Lammer et al. 2009). Oligonucleotides were synthesized through Integrated DNA Technologies (IDT).

**Table 1:** Four MLPA probe sequences targeting three *ISL1* exons

Exon	Left probe	Right probe
E1	CCAATGGCGATGGAGCTGAGTTGGAGCAGAGAAGTTT	GAGTAAGAGATAAGGAAGAGAGGTGCCCGAGCCGCGC
E5	GCTTACAGGCTAACCCAGTGGAAGTAC	AAAGTTACCAGCCACCTTGGAAAGTAC
E6_1	TTTTTCAGAAGGAGGACCGGGCTCTAATTCCACTGGCAGTGAAGTAG	CATCAATGTCCTCTCAACTTCCAGATACACCTAACAGCATGGTAGCC
E6_2	GCATTGCAACAAGGTTACCTCTATTTTGCCACAAGCGTCTCGGGA	TTGTGTTTGACTTGTGTCTGTCCAAGAACTTTTCCCCCAAAGATG

Only target-specific probe sequences are listed. When the probes were synthesized, universal primer annealing sites were attached to 5′-end of left probe and to 3′-end of right probe, respectively.

### MLPA reaction and data analyses

We used ∼20 ng genomic DNA and followed MLPA protocol obtained from MRC-Holland for annealing, ligation, and PCR steps. PCR products were labeled with FAM dye using the primers from the SALSA MLPA EK1 reagent kit (MRC-Holland, Amsterdam, the Netherlands). Size standard GeneScan-LIZ 600 (Life Technologies) was mixed with probes in formamide (Life Technologies) according to the MRC-Holland protocol. The probe and size standards were heat denatured and loaded into a capillary electrophoresis system (3730XL DNA Analyzer, Life Technologies) performed at Quintara Biosciences. Electropherograms were manually reviewed in GeneMapper software (Life Technologies) for quality assessment and the data analyses were subsequently performed using SeqPilot MLPA software (JSI medical systems, Kippenheim, Germany). The SeqPilot MLPA software normalizes the peak pattern of all 14 probes within samples and then compares to other samples. Control DNA was included in 6 wells in each 96-well plate for quality control and data normalization. When control sample replicates were discordant, data were discarded and the experiments were repeated. Control peaks from the control DNA were assigned a dosage quotient (DQ) of 1.0. DQ analysis is a standard method of interpreting MLPA data and consists of calculating the ratio of sample and reference peak area (Yau et al. 1996). A DQ of 0.5 was expected for heterozygous deletion cases and a DQ of 1.5 was expected for heterozygous duplication cases. DQ ranging between 0.65 and 1.35 was deemed to have no copy number variation present. We selected samples with DQ measurements below 0.65 or above 1.35 thresholds for the follow-up confirmatory experiments.

### Array comparative genomic hybridization

To confirm our findings from the MLPA experiments, we performed high-resolution array-CGH. We used microarrays containing 963,029 distinct features, 1000 replicated features and Internal Quality Control Features (Agilent Technologies (Santa Clara, CA), SurePrint G3 Human CGH Microarray Kit, 1 × 1 M Cat No. G4447A). Test and reference WGA-DNA was labeled with Cy3 and Cy5 dyes using the random priming procedure according to the manufacture's instruction (Agilent Technologies, SureTag Complete DNA Labeling Kit, Cat No. 5190-4240). We performed a dye-swap experiment to confirm the reproducibility of the deletion of *ISL1*. For the dye-swap experiments, test and reference WGA-DNA was labeled with Cy5 and Cy3, respectively. The test and reference probes were combined in hybridization solution containing Cot-I DNA, denatured, and incubated at 37°C to allow Cot-I DNA to hybridize to repetitive DNA sequences. The probes were then applied to the slide surface and hybridized at 65°C in a rotating hybridization oven for 48 h. Slides were washed according to the manufacturers' recommended protocols (Agilent Technologies, Oligo aCGH/ChIP-on-chip Hybridization Kit, Cat # 5188-5220). Hybridized slides were imaged using a G2565CA Microarray Scanner System (Agilent Technologies) and processed for feature extraction. The extracted data were normalized and displayed graphically by plotting chromosome positions (X axis) against log_2_(Test/Reference) values (Y axis) using CytoGenomics Edition 2.0.6.0 software (Agilent Technologies).

### Genotyping, breakpoint junction cloning, and sequencing

We designed 23 PCR primer sets containing 34 known SNPs with minor allele frequency (MAF) >10% within a 44-kb genomic region spanning 19-kb upstream from the 5′-end of *ISL1* mRNA starting nucleotide to 13-kb downstream from the 3′-end of *ISL1* mRNA. PCR primers were designed using Primer3 software (Rozen and Skaletsky 2000). The average spacing between SNPs resulted in 1.2 kb (Table S1). PCR was performed using FastStart High Fidelity PCR System (Roche Cat. No. 04 738 292 001) according to the manufacture's instruction. Prior to sequencing, PCR products were treated with ExoSAP-IT (USB Corp., Cleveland, OH Cat. No. 78200) to enzymatically remove unincorporated dNTPs and excess primers. As per manufacture's protocol, 5 *μ*L of PCR product was mixed with 2 *μ*L ExoSAP-IT and incubated at 37°C for 15 min, followed by an inactivation step of 80°C for 15 min. PCR products were sequenced using the Sanger method with the 3730XL DNA Analyzer (Life Technologies) at Quintara Biosciences. The region was expanded to both sides to find informative heterozygous SNPs by adding four more SNPs: rs6449586 to the centromeric side, and rs6449622, rs4865664, and rs11951998 to the telomeric side. We visually genotyped SNPs using Sequencher software (Gene Codes Co.).

Using the same PCR primers used for genotyping, we rearranged the primer combinations so that we could amplify a genomic DNA fragment that contained the deletion breakpoint junction (Table 2). Amplification was performed using the Expand Long Template PCR System (Roche Cat. No. 11681842001). A 50-*μ*L PCR was set up according to the manufacturer's recommended protocol: 300 ng of WGA-DNA, 5 *μ*L of Buffer 1 (for 0.5–9 kb size fragments), 350 *μ*mol/L each dNTP, 300 nmol/L primers, 0.75 *μ*L (3.75 units) Expand Long Template Enzyme Mix. The PCR was performed using the following cycles on a DNA engine thermal cycler (MJ Research–BioRad, Hercules, CA): 93°C 2 min; 10 cycles: 93°C 10 sec, 60°C 30 sec, 68°C 2 min; 25 cycles: 93°C 15 sec, 60°C 30 sec, 68°C 2 min plus 20-sec cycle elongation for each successive cycle; 68°C 7 min for final extension. PCR products were electrophoresed on 1% agarose gels, and visualized with ethidium bromide staining. DNA size marker (All-purpose LO DNA Marker, 50–2000 bp, Bionexus, Oakland, CA) was loaded in the gel.

**Table 2:** PCR primer combinations used to amplify deletion breakpoint junction

PCR	Primer ID	Primer F sequence	Primer ID	Primer R sequence	Distance (kb)	Amplicon size (kb)
1	5p1-F	aaacgggaaaggggatacat	3p3-R	ttgcccacacctaggtaaaga	21.5	1.3
2	5p1-F	aaacgggaaaggggatacat	3p2-R	acatcatggaagccttggtc	20.5	No
3	5p1-F	aaacgggaaaggggatacat	3p1-R	ccacctggatttggaagaaa	19.2	No

These PCR primer sets were used to amplify a genomic DNA fragment that could only be created by the deletion event.

The 1.3-kb PCR fragment containing the deletion breakpoint junction was cloned into a pCR2.1-TOPO cloning vector using the TOPO TA Cloning Kit (Life Technologies), and transformed into NEB Turbo Competent *E. coli* (New England Biolabs, Ipswich, MA) at Quintara Biosciences. The insert DNA was sequenced using the Sanger method with a 3730XL DNA Analyzer (Applied Biosystems) performed by Quinatara Biosciences. After the initial sequence from T7 priming site, two internal primers (ACCTAGGTGTGGGCAATTTTT and CTTGTGCAGGTTCTATTCTGTGA) were designed to obtain reverse strand sequences around the breakpoint junction region and to read through the entire fragment.

The DNA sequences were aligned to the human genome sequence (hg19) using the Blat sequence alignment tool from the UCSC Genome Browser. The deletion break points were determined from the DNA sequence alignment results.

### Sequencing ISL1 exons

We designed PCR primers that amplify each exon of *ISL1*. The exons were defined using UCSC Genes Track Settings on UCSC Genome Browser. The exon sequences containing ± 500 bp intronic sequences were downloaded from the UCSC Genome Browser. The SNPs, Segmental Duplications and Repeat Maskers Tacks were activated and used to mask these sequences to avoid from the primer annealing sites. Most of the PCR primer sets were designed on intronic sequences surrounding exons using online Primer3 software (Rozen and Skaletsky 2000). The PCR fragment size were set to be 500–600 bp, the optimal size range for GS FLX Titanium sequencing system (Roche 454). If it was not possible to design the suitable primer combinations, then we set the fragment size to be 350–800 bp as a secondary choice. For exons 1 and 6 that are larger than 400 bp, we designed overlapping amplicons. PCR primers are listed in Table S2. We used Access Array system which provides parallel amplification of 48 sample inputs with 48 primer set inputs to make all possible 2304 combinations of samples and primer sets using Integrated Fluidic Circuit PCR system (Fluidigm Corp., South San Francisco, CA). Fluidigm-specific adaptor sequences (ACACTGACGACATGGTTCTACA, TACGGTAGCAGAGACTTGGTCT) were added to the 5′-end of forward and reverse target-specific PCR primers, respectively, when oligonucleotides are synthesized. These adaptor sequences enable direct attachment of barcode sequences and GS FLX sequencer-specific tags (454A and 454B) to the PCR products. The sample-specific barcode sequence assigns a sample identity to each DNA sequence read. PCR primers were validated using the protocol provided by Fluidigm. PCR products were analyzed using a Bioanalyzer system (Agilent Technologies) to estimate the fragment size and approximate DNA concentration of each amplicon. The validated PCR primers were subsequently used for PCR on the Access Array system, and sequenced using GS FLX+ DNA sequencing instrument (Roche 454). The resulting DNA sequences were aligned against the reference genomic DNA sequences using SeqNext software (JSI medical systems). Novel variations which are not registered in SNP database (dbSNP) were confirmed by the Sanger DNA sequencing method. If the variations were located within coding sequences and resulted in nonsynonymous amino acid changes, we further investigated the potential functional consequences using PolyPhen-2 (Adzhubei et al. 2010), SIFT (Kumar et al. 2009), PROVEAN (Choi et al. 2012), and SNAP (Bromberg and Rost 2007) online software.

## Results

### Identifying a deletion of ISL1 using MLPA

**Figure 1:**
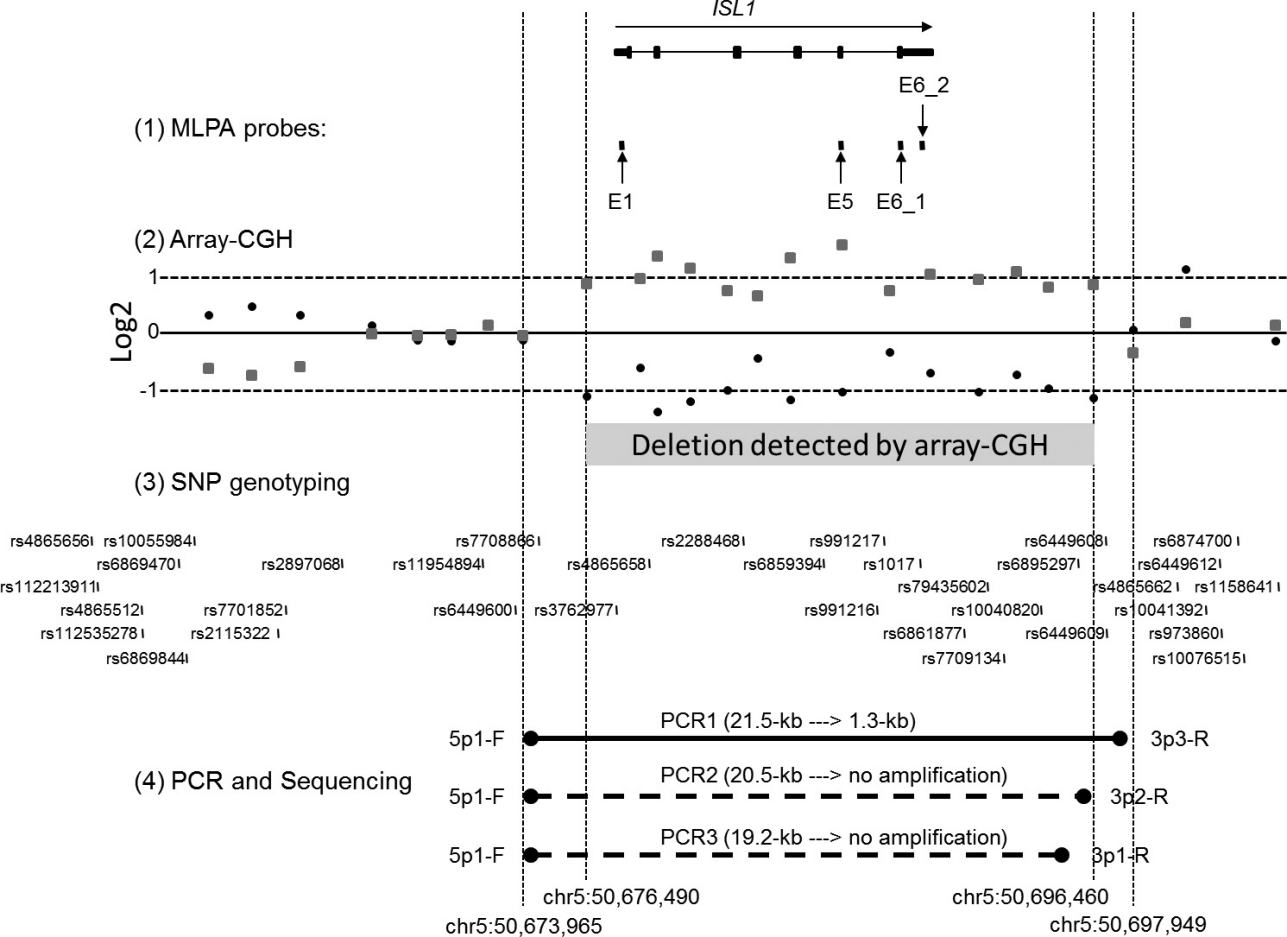
Genomic organization of *ISL1* and 20-kb deletion. The genomic organization of the six exons of human *ISL1* is shown at the top of the Figure. (1) Four MLPA probes were designed to amplify exons 1, 5, and 6 and are designated by arrows as E1, E5, E6_1, and E6_2, respectively. Each exon showed a loss of copy number by MLPA (data not shown). (2) Two array-CGH experiments with Agilent 1 million features microarrays were performed: hybridization with Cy3-labeled test versus. Cy5-labeled reference DNA (Log_2_ values plotted by black circles) and Cy5-labeled test versus. Cy3-labeled reference probes (“dye swap” whose Log_2_ values are plotted by gray squares). The Log_2_(Test/Reference) values were plotted for each microarray oligonucleotide feature at each chromosomal locus. Negative Log_2_ values of 14 consecutive probes (black circles) are seen in a zoomed-in array-CGH plot encompassing *ISL1* on chromosome 5, indicating a deletion. A nearly mirror image of these Log_2_ values is observed from the dye-swap experiment (gray squares). The two outside dashed vertical lines indicate the positions of the innermost oligonucleotide features that showed normal Log_2_ values. The two inner dashed vertical lines show the positions of the two outermost of the 14 oligonucleotide features that showed negative Log_2_ values. (3) Thirty-four genotyped SNPs are shown relative to the genomic organization of *ISL1*. (4) We designed several PCRs to identify the chromosomal breakpoints. PCR1: a 21.5-kb product would be expected from the normal chromosome 5 using a primer set, 5p1-F and 3p3-R, indicated by black circles, and whose sequences are listed in Table 2. A 1.3-kb amplicon was generated (see also Figure 2 lane 2) because these primers were brought into proximity of each other by the deletion of the intervening 20-kb chromosomal segment. PCR2 and 3: No PCR product was generated using the same forward primer (5p1-F), in combination with two different reverse primers, 3p2-R and 3p1-R, located inside the right pair of vertical dashed lines. This indicates that these two reverse primers are located within the deleted region.

We screened 225 and 164 DNA samples from subjects with TOF or d-TGA, respectively, using four MLPA probes designed to interrogate *ISL1* exons 1, 5, and 6 (Table 1 and Fig. 1). We identified deletion signals (DQ < 0.5) from each of four *ISL1* MLPA probes from one female infant born with isolated d-TGA and intact ventricular septum (data not shown). She was born to 31-year-old G_2_P_2_ Hispanic mother after 40 weeks of uncomplicated pregnancy. There was no maternal diabetes, epilepsy, or other medical condition requiring treatment during her pregnancy. She weighed 2948 g and had no associated dysmorphic facial features and no cardiac arrhythmias. There is no family history of any individual with cardiac defect or other major malformation.

### Array comparative genomic hybridization

In order to confirm the microdeletion identified from the MLPA experiments, and to narrow the deletion breakpoints, we performed array-CGH experiments using a 1 million feature oligonucleotide microarray. A 19,971 bp deletion (chr5:50,676,490-50,696,460) was identified at chromosome 5q11.1 that affected 14 oligonucleotide features (Fig. 1). We repeated the experiment by swapping the dyes for labeling reference and test probes and found nearly identical mirror-image log_2_ ratio values (Fig. 1). The most centromeric position of the oligonucleotide sequence within the deleted region starts at chr5:50,676,490 while the next oligonucleotide feature outside the deleted region ends at chr5:50,673,965. The centromeric side of the breakpoints was, thus, narrowed within 2524 bp or chr5:50,673,966-50,676,489. Similarly, the most telomeric position of the oligonucleotide sequence within the deleted region ends at chr5:50,696,460 while the next oligonucleotide feature outside the deleted region starts at chr5:50,697,949. The telomeric side of the breakpoints was, therefore, limited within 1489 bp or chr5:50,696,461-50,697,949.

We searched copy number variations (CNVs) containing *ISL1* registered in DECIPHER (Firth et al. 2009), DGV (Macdonald et al. 2014), and The International Standards for Cytogenomic Arrays (ISCA) Consortium databases, but no identical microdeletion was found in these databases.

### Genotyping single-nucleotide polymorphisms

To provide additional evidence of this microdeletion, we genotyped 34 known SNPs within a 44-kb genomic region encompassing *ISL1*. We hypothesized that hemizygous SNP genotypes would be found within the deleted region, and heterozygous SNPs would be found flanking the deleted region. We designed 23 PCR primer sets to amplify genomic DNA fragments containing the 34 SNPs (Table S1). The PCR products were sequenced by the Sanger DNA sequencing method. No heterozygous SNP allele was found within the predicted deleted region, supporting the array-CGH results. We further walked out 25 kb from the most centromeric SNP (rs4865656), and found a heterozygous SNP (rs6449586) (Table S1). We walked out 33 kb from the most telomeric SNP (rs1158641), but no heterozygous SNPs were found in the 38-kb region telomeric of the other deletion breakpoint (as determined by array-CGH). As a result, we found no heterozygous SNPs spanning a >76-kb genomic region. The result was unexpected, considering that the array-CGH results predicted only a 20-kb microdeletion. We reviewed linkage disequilibrium data for the ethnic group of this subject using the genome variation server (GVS), and found strong linkage disequilibrium across the corresponding genomic region around *ISL1*. It is most likely that the highly homozygous genomic region represents a large haplotype block, found in several ethnic groups.

### PCR confirmation and sequencing

**Figure 2:**
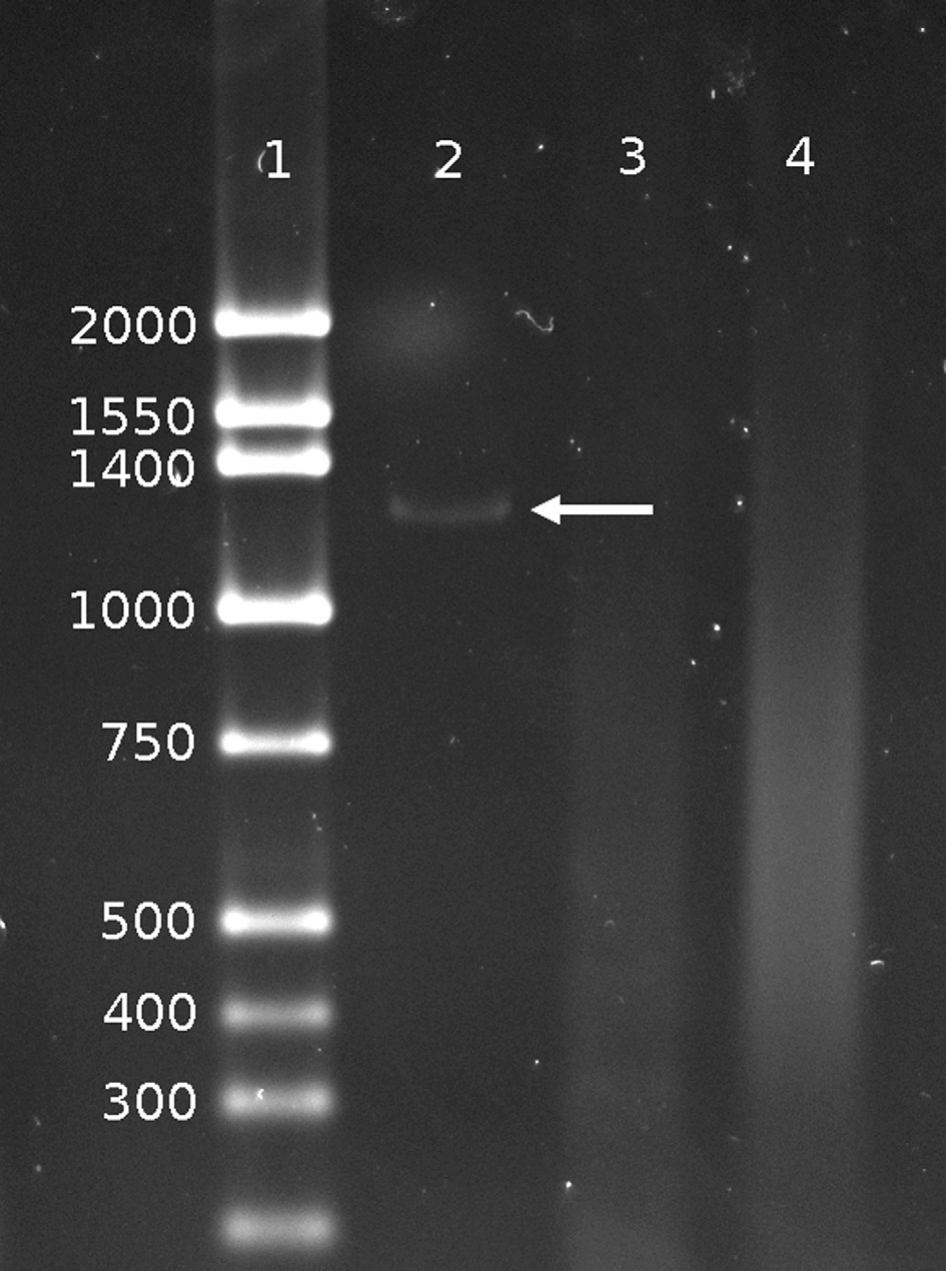
PCR amplification across deletion breakpoint junction. Lane 1: DNA size marker ladder is shown with their sizes (bp). Lane 2: A 1.3-kb PCR product indicated by an arrow is generated using primers 5p1-F and 3p3-R (Table 2) with DNA isolated from the subject. Lane 3: No amplification was seen using primers 5p1-F and 3p1-R with the subject DNA indicating that primer 3p1-R is located within the deleted region. Lane 4: No PCR product was generated using the same primer set as in Lane 2 (5p1-F and 3p3-R) but, with control DNA which has no deletion.

The array-CGH experiments narrowed the deletion breakpoints within 2.5-kb centromeric and 1.5-kb telomeric regions of *ISL1*. We designed a PCR to amplify a genomic DNA fragment that would contain the deletion breakpoint junction. We made a new pair of PCR primers for PCR1: 5p1-F and 3p3-R (Table 2). The distance between these primers in the reference genome is ∼21.5-kb, too large to amplify by standard PCR procedure. As expected, no PCR product was generated using these primers and control DNA (Fig. 2, Lane 4). In contrast, when we amplified genomic DNA from our subject using the PCR1 primers, a distinct 1.3-kb PCR product was observed (Fig. 2 Lane 2). These PCR primers could only have been brought into close enough proximity to permit PCR to precede if the intervening 20-kb chromosomal segment was deleted (Fig. 1). To further verify the breakpoint information obtained by array-CGH, we performed two additional PCRs that used the same centromeric primer 5p1-F plus telomeric primers located within the putative deleted segment (PCR2: 5p1-F and 3p2-F, and PCR3: 5p1-F and 3p1-F) (Table 2). No PCR product was generated (Fig. 2, Lanes 3) indicating these two reverse primers must be located within the deleted region (Fig. 1).

**Figure 3:**
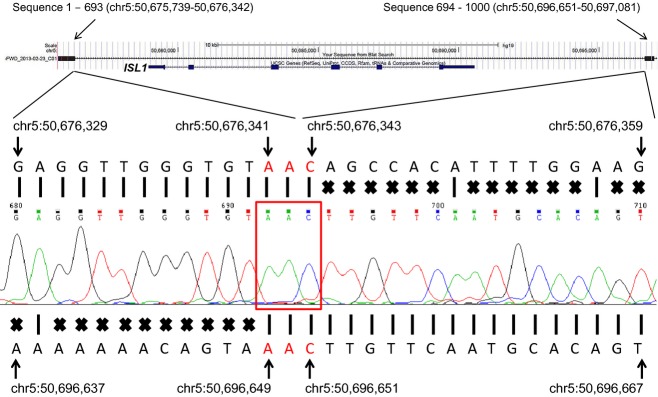
DNA sequence across deletion breakpoint junction. A 1000 base DNA sequence from centromeric end of the 1.3-kb fragment was aligned using the Blat DNA sequence alignment tool from the UCSC Genome Browser (Top). *ISL1* with 6 exons is shown in the Browser view. The DNA sequence was split into two parts and mapped onto two genomic loci indicated by black rectangles at both sides of the Browser view. The first 693 bases (sequence 1–693) mapped to chr5:50,675,739-50,676,342, and the following 307 bases (sequence 694–1000) localized to chr5:50,696,651-50,697,081. The electropherogram is shown in the middle of the figure with base calls shown in small colored letters directly above the electropherogram. Reference hg19 genomic DNA sequence at chr5:50,676,329-50,676,359 is presented above the electropherogram. Perfect DNA sequence alignments indicated by the vertical bars are seen at chr5:50,676,329-50,676,343. Sequence mismatches are depicted by “X” marks followed by the perfect alignments. Reference hg19 genomic DNA sequence at chr5:50,696,637-50,696,667 is shown at the bottom. Perfect DNA sequence alignments are observed at chr5: 50,696,649-50,696,667 after sequence mismatches (depicted by “X” marks). Genomic hg19 positions are indicated with arrows for some nucleotides. The sequence result confirms the “hybrid” DNA sequence which is generated by a 20,308 bp deletion via nonhomologous end joining mechanism. A microhomologous DNA sequence, “AAC”, surrounded by the red rectangle, is found at the junction point from both proximal and distal DNA fragments.

We cloned the 1.3-kb fragment into a vector using the TOPO cloning procedure and sequenced the entire fragment. DNA sequences were mapped against the hg19 reference human genome sequence using the Blat DNA sequence alignment tool from the UCSC Genome Browser (Kent 2002). As expected, the DNA sequence mapped to two genomic loci (Fig. 3). From the sequence alignment of 1000 base sequences from one end of the clone against the reference genome, the first 693 bases (sequence 1–693) mapped to chr5:50,675,739-50,676,342, and the following 307 bases (sequence 694–1000) localized to chr5:50,696,651-50,697,081 (Fig. 3). The DNA sequence alignment revealed that the size of the deletion is 20,308 bp, encompassing the entire coding region of *ISL1*. A three base-pair, “AAC,” microhomology sequence was found at the breakpoint junction (Fig. 3). The microhomology sequence indicates that the deletion was caused by random DNA breakage, and repaired by a nonhomologous end joining mechanism (Pastwa and Blasiak 2003).

### Sequencing ISL1

We sequenced most exonic and partial flanking intronic sequences of *ISL1*. It was not possible to design a suitable PCR primer pair amplifying a fragment containing bases 1–98 of exon4, due to a presence of 87 bp repetitive sequences adjacent to 5′-end of exon4 and high GC content surrounding exon4. We identified 18 known SNPs that were registered in dbSNP, and nine novel variants (Table S3). We found two nonsynonymous mutations: (1) rs200172777 [chr5:50683541 (hg19), C->A] that causes a P146T amino acid change; and (2) rs121912286 [chr5:50685756 (hg19), A->G] that results in N252S. We genotyped our matched controls, and reviewed the genotype frequencies from the Exome Variant Server database and 1000 Genome Database. The genotype frequencies for rs200172777 are AA =0, AC = 2, and CC = 4195 in a European American population, AA = 0, AC = 2, and CC = 2063 in an African American in the Exome Variant Server database, and AA = 0, AC = 3, and CC = 355 in our California control population. The allele frequencies in 1000 Genomes Database are A = 0.0005/C = 0.9995. Similarly, the genotype frequencies for rs121912286 are GG = 0, GA = 3, and AA = 4286 in European Americans, GG = 0, GA = 2. and AA = 2183 in African Americans in the Exome Variant Server, GG = 0, GA = 3, and AA = 353 in our California control population. No G allele was found in the 1000 Genomes Database. We further investigated these two amino acid changes using PolyPhen-2, SIFT, PROVEN, and SNAP. PolyPhen-2 indicated “possibly damaging” for P146T, but SIFT, PROVEN, and SNAP designated “neutral”, “tolerated”, and “neutral”, respectively. Similarly, PolyPhen-2, SIFT, PROVEN, and SNAP analysis for N252S indicated “benign”, “neutral”, “tolerated”, and “neutral”, respectively. It seems unlikely that either P146T or N252S has adverse functional consequences to ISL1 that might induce conotruncal heart defects, based on these computational analyses. Four and three known SNPs are located in 5′-UTR and 3′-UTR, respectively. The remaining nine known SNPs are located in intronic sequences. All of the nine unknown variants were located in noncoding sequences. Of these unknown variants, two and one unknown SNPs are found in 5′-UTR and 3′-UTR, respectively, and the remaining six SNPs are located in intronic sequences. We mapped back these known and unknown SNPs on UCSC Genome Browser using “custom track” function, and aligned against “Integrated Regulation from ENCODE tracks” (Figure S1). Figure S1 shows that some SNPs discovered are located within the putative regulatory DNA sequences.

## Discussion

We screened 389 infants born with conotruncal defects for microdeletions and DNA sequence variation of *ISL1*. From an infant with isolated d-TGA, we found a novel 20-kb microdeletion encompassing the entire *ISL1* coding region, but excluding the flanking genes. Detailed molecular characterization of the deletion breakpoint junction revealed no repetitive DNA sequences or segmental duplications surrounding *ISL1*, but we found a microhomology at the deletion breakpoint junction. We conclude that the microdeletion was caused by random chromosomal breakage and repaired by a nonhomologous end joining mechanism (Pastwa and Blasiak 2003). This subject's single copy of *ISL1* showed normal exonic sequences. Unfortunately, parental DNA was not available and it was not possible to determine whether the deletion was de novo or inherited. Because the murine homozygous KO of *Isl1* induces conotruncal defects, however, we suspect that haploinsufficiency of *ISL1* caused our subject's nonsyndromic d-TGA.

From sequencing exons of *ISL1*, we identified two known rare SNPs that result in nonsynonymous amino acid changes. The first rare variant, rs121912286, results in N252S change and causes gain of function, which could potentially lead to greater activation of downstream targets, such as myocyte enhancer factor 2C (MEF2C), involved in cardiac development (Friedrich et al. 2013). Those investigators found one subject born with dilated cardiomyopathy who was homozygous for N252S, but no heterozygous carriers in the extended family had a conotruncal heart defect. Since we found that nearly 1% of nonmalformed California controls were heterozygous for N252S, it is not clear that this variant is associated with risk for conotruncal defects. The second rare variant that we found, rs200172777, causes a P146T change, and has not been reported from any subject with a conotruncal defect. Similarly, we found 1% of nonmalformed California controls were heterozygous for this rare variant. In addition, we found that several SNPs are located within “DNaseI Hypersensitivity Clusters” and “Transcription factor binding sites”. Further biological investigations are required to determine whether these SNPs have functional consequences. Recently, exons of *ISL1* were sequenced from 256 Japanese subjects with nonsyndromic cardiac outflow tract defects including 125 subjects with TOF, but no DNA sequence variation in *ISL1* was identified (Kodo et al. 2012). Based on our combined DNA sequencing results, mutations of coding regions of *ISL1*, if they cause conotruncal defects, are uncommon.

*ISL1* is a member of the LIM/homeodomain family of transcription factors and it encodes a protein that binds to the enhancer region of the insulin gene (Tanizawa et al. 1994). Homozygous *Isl1* knockout mice showed embryonic hearts that were essentially missing segments, that is, lacking right ventricles and outflow tracts, resembling some conotruncal heart defects in humans (Cai et al. 2003). Genetic susceptibility for human congenital heart defects was recently associated with common genetic variants of *ISL1*, but the study population included a very broad spectrum of heart defects, limiting the applicability of the results (Stevens et al. 2010). They reported that the T allele for rs1017 within the 3′-UTR was associated with congenital heart defects. We calculated T allele frequency of rs1017 to be 0.37 from our sequencing results (Table S3). We observed the nearly identical T allele frequency (T = 0.3669) from 1000 Genomes Database. Although we did not genotype our California controls, it seems unlikely that the T allele of rs1017 is associated with either d-TGA or TOF.

While *ISL1* has been considered as a candidate gene for human heart defects, no mutation associated with any cardiac defect has been identified. Recently, a 601-bp duplication of part of exon6 and 3′-UTR of *ISL1* [chr5:50,689,340-50,689,940 (hg19)] was identified using exon capture-based sequencing strategy from a subject with TOF (Bansal et al. 2014). It is, however, not clear that this partial duplication of exon 6 of *ISL1* had any functional consequence that might have led to TOF. We screened DNA samples obtained from 389 subjects with TOF or d-TGA for microdeletions/duplications using 32k bacterial artificial chromosome (BAC) array-CGH, but did not find a deletion/duplication of *ISL1* (Osoegawa et al. 2014). The 32k BAC array-CGH lacks the resolution to identify a 20-kb deletion. The 105k features microarray that was designed by the ISCA Consortium, and which is widely used for clinical diagnostic testing, has only four oligonucleotide features within the 20-kb region of *ISL1*. Thus, this microdeletion could have been missed with routine array-CGH clinical testing. The resolution of the 105k microarray is closer to 100 kb in size when three consecutive features were grouped to identify microdeletions/duplication. Although it was possible to identify the 20-kb deletion using a 1 million feature oligonucleotide microarray, the cost of this dense microarray are prohibitive for screening genome-wide copy number changes from large populations or for routine clinical diagnostic testing. MLPA is an alternative, powerful, and cost-effective approach to identifying microdeletions/duplications smaller than 100 kb involving known candidate gene loci. In theory, it is feasible to identify even a single exon deletion using MLPA, if the MLPA probes are located within a deleted exon.

In this large-scale population-based study, we found a subject with isolated d-TGA who had a 20-kb deletion encompassing the entire coding region of *ISL1*. This is the first report of a mutation of *ISL1* associated with a conotruncal heart defect and adds to the list of transcription factors for which copy number variation adversely influences heart development.

## Supporting Information

**Table S1.** PCR primers used for genotyping. **Table S2.** PCR primers used for sequencing exons of ISL1. **Table S3.** SNPs identified from sequencing ISL1. **Figure S1.** Distribution of SNPs identified by sequencing.
