# Supplementary material for: Haploinsufficiency of insulin gene enhancer protein 1 (ISL1) is associated with d-transposition of the great arteries
Source: Mol Genet Genomic Med. 2014 Apr 17;2(4):341–51. doi: 10.1002/mgg3.75 (PMC4113275; doi:10.1002/mgg3.75)
Supplement: Supplementary file 1 [file mgg30002-0341-SD1.docx]

Supplemental Table I: PCR primers used for genotyping

| SNP ID | Genotype | chr5: (hg19) | Primer ID | Forward primer | Reverse primer | MAF |
| --- | --- | --- | --- | --- | --- | --- |
| rs6449586 | T/C | 50634878 | 5p15 | gagaacaacgaaggctccag | tcagcctctcttgcctcaat | 0.48 |
| rs4865656 | G | 50659788 | 5p8 | tgctggatcccttaaccttg | tgccgggctaatttttgtat | 0.41 |
| rs112213911 | C | 50659982 | 5p8 | tgctggatcccttaaccttg | tgccgggctaatttttgtat | 0.15 |
| rs4865512 | A | 50661601 | 5p7 | ctccatctccccagtaccaa | gcctgttcttgtaccttcatttc | 0.37 |
| rs112535278 | C | 50661672 | 5p7 | ctccatctccccagtaccaa | gcctgttcttgtaccttcatttc | 0.50 |
| rs6869470 | C | 50663015 | 5p6 | aaatcagggctgggagaact | aaagggaatatgggctgctt | 0.39 |
| rs6869844 | C | 50663234 | 5p6 | aaatcagggctgggagaact | aaagggaatatgggctgctt | 0.18 |
| rs10055984 | C | 50663550 | 5p6 | aaatcagggctgggagaact | aaagggaatatgggctgctt | 0.19 |
| rs2115322 | A | 50666618 | 5p5 | ctgaccaacagccaacaaga | ggagacagacaactggcaca | 0.41 |
| rs7701852 | G | 50666897 | 5p5 | ctgaccaacagccaacaaga | ggagacagacaactggcaca | 0.15 |
| rs2897068 | G | 50668976 | 5p4 | gcctttcccatctacccttc | ggaggttttgtttagttttgttgg | 0.23 |
| rs11954894 | C | 50674131 | 5p3 | aactgaccgggcatttactg | aacgggcagctctgagttta | 0.19 |
| rs6449600 | C | 50675297 | 5p2 | ccagccgcttttgataagtc | ggtggtggcttattgaggaa | 0.17 |
| rs7708866 | T | 50676126 | 5p1 | aaacgggaaaggggatacat | cctgcacaaacatcatgctc | 0.19 |
| rs3762977 | A | 50679014 | E1-5 | aggggacagaaggaagagga | cctgtgcctgagagagaacc | 0.11 |
| rs4865658 | G | 50680233 | E1-6 | ggttctctctcaggcacagg | attccaaatccggagaaacc | 0.37 |
| rs2288468 | T | 50683655 | E3-4 | cagcaagaacgacttcgtga | acagcgcaaattgcatacag | 0.39 |
| rs6859394 | T | 50686582 | E4-7 | ttggattcctgcctacatcc | caaagcatttgggtctggtt | 0.37 |
| rs991216 | C | 50688533 | E6-1 | agtgggtgcagaggctagaa | acatgttgccctgggagtag | 0.65 |
| rs991217 | G | 50688801 | E6-1 | agtgggtgcagaggctagaa | acatgttgccctgggagtag | 0.35 |
| rs1017 | A | 50690095 | E6-3 | aacagcatggtagccagtcc | cgcttgtggcaaaatagagg | 0.37 |
| rs6861877 | G | 50691723 | E6-10 | cccaggatgatgctttgact | agtgggtccagtgttttcca | 0.37 |
| rs79435602 | T | 50692601 | E6-11 | aagtgaggcacccatctttg | ggtgaatagcagccaagcat | 0.10 |
| rs7709134 | G | 50693176 | E6-11 | aagtgaggcacccatctttg | ggtgaatagcagccaagcat | 0.42 |
| rs10040820 | A | 50694579 | 3p1 | ccacctggatttggaagaaa | tgtttggattagccctccag | 0.35 |
| rs6895297 | G | 50695877 | 3p2 | acatcatggaagccttggtc | attttcgctgtcaccaaacc | 0.37 |
| rs6449608 | T | 50696922 | 3p3 | ttgcccacacctaggtaaaga | cagccgttccatgttagtga | 0.35 |
| rs6449609 | T | 50696967 | 3p3 | ttgcccacacctaggtaaaga | cagccgttccatgttagtga | 0.35 |
| rs4865662 | A | 50699552 | 3p5 | gccaggctgatcttgaactc | tataaggggctttcggaaca | 0.39 |
| rs10041392 | A | 50700597 | 3p6 | gtatgccaggctcccttaca | ggctagggaaatgcaacaaa | 0.37 |
| rs6449612 | C | 50701102 | 3p7 | ttgtggtgcattcaaagaaca | caaaactcaaacacacagcttca | 0.37 |
| rs973860 | C | 50701139 | 3p7 | ttgtggtgcattcaaagaaca | caaaactcaaacacacagcttca | 0.35 |
| rs6874700 | T | 50701750 | 3p8 | aaagctgtgccccatacatc | agcatagtggcaacgaaacc | 0.32 |
| rs10076515 | G | 50702024 | 3p8 | aaagctgtgccccatacatc | agcatagtggcaacgaaacc | 0.37 |
| rs1158641 | C | 50703285 | 3p9 | cattcagtgttcacccatgc | aaaggtgccccaaaagttct | 0.38 |
| rs6449622 | C | 50709369 | 3p12 | attcccccagagagcaaaat | catccagcaggtacacatgg | 0.37 |
| rs4865664 | A | 50722238 | 3p14 | ggaagcacctcttcatcgtc | ggaatgaaccccagtgattg | 0.40 |
| rs11951998 | T | 50736314 | 3p20 | cagagctggtcctctgaacc | cacccctgtattggaggaga | 0.39 |

These PCR primer sets were used to amplify genomic DNA fragment. The resulting PCR products were sequenced by the Sanger sequencing method.

Supplemental Table II: PCR primers used for sequencing exons of *ISL1*

| Exon | Size (bp) of exon | Forward | Reverse | PCR product size (bp) |
| --- | --- | --- | --- | --- |
| 1 | 576 | gggacctcgtcaacaggtag | CTGCTCCAACTCAGCTCCAT | 458 |
| 1 | 576 | agacctacCCACCAGCCACT | CCTCGCGGATCTAGGAGTC | 559 |
| 1 | 576 | GGAGCTGAGTTGGAGCAGAG | cctgtgcctgagagagaacc | 385 |
| 2 | 190 | aaaatgagcgggtttgattg | gtggggagattcagggaaat | 524 |
| 3 | 260 | aagtgaccccctcttcctgt | ggggaatcaagggagacagt | 527 |
| 4 | 287 | CTGTGCTGAACGAGAAGCAG | ctccggttcacaggtacagg | 508 |
| 5 | 168 | aggtacggcggattaactga | ttttccctgcatttgtctcc | 477 |
| 6 | 1236 | cagtgtcctttatttatttctcaacc | TGTGCAGTGAAATGAAAATGC | 492 |
| 6 | 1236 | AACAAAACGCAAAACCCAGT | CGCTTGTGGCAAAATAGAGG | 516 |

The primers with lower case or capital letters indicate that these primers are located on introgenic or exonic sequences, respectively. For exons 1 and 6, we designed overlapping amplicons.

Supplemental Table III: SNPs identified from sequencing *ISL1*

| Genome position  hg19 | SNP ID | Homo | Het | AA change | Loci | 1000 Genome |
| --- | --- | --- | --- | --- | --- | --- |
| chr5:50,679,014 | rs3762977 | 0 | 3 |  | 5’-UTR | G=0.1056  A=0.8944 |
| chr5:50,679,204 | rs36216897 | 0 | 1 |  | 5’-UTR | G=0.0165  A=0.9835 |
| chr5:50,679,266 | rs116222082 | 0 | 2 |  | 5’-UTR | A=0.0092  G=0.9908 |
| chr5:50,679,292 | unknown | 0 | 1 |  | 5’-UTR |  |
| chr5:50,679,415 | unknown | 0 | 1 |  | 5’-UTR |  |
| chr5:50,679,459 | rs3917084 | 0 | 2 |  | 5’-UTR | G=0.0119  A=0.9881 |
| chr5:50,679,550 | rs150104955 | 0 | 3 |  | Intron1 | T=0.0147  C=0.9853 |
| chr5:50,680,144 | unknown | 0 | 2 |  | Intron1 |  |
| chr5:50,680,233 | rs4865658 | 79 | 148 |  | Intron1 | A=0.3646  G=0.6354 |
| chr5:50,680,249 | rs4151674 | 1 | 23 |  | Intron1 | G=0.0257  A=0.9743 |
| chr5:50,683,309 | rs17847230 | 0 | 3 |  | Intron2 | G=0.0119  C=0.9881 |
| chr5:50,683,541 | rs200172777 | 0 | 1 | P146T | Exon3 | A=0.0005  C=0.9995 |
| chr5:50,683,655 | rs2288468 | 64 | 148 |  | Intron3 | C=0.3669  T=0.6331 |
| chr5:50,685,756 | rs121912286 | 0 | 1 | N252S | Exon4 | G=0.0000  A=1.0000 |
| chr5:50,685,921 | unknown | 0 | 1 |  | Intron4 |  |
| chr5:50,686,009 | unknown | 0 | 1 |  | Intron4 |  |
| chr5:50,687,005 | unknown | 0 | 1 |  | Intron4 |  |
| chr5:50,687,007 | rs189872668 | 0 | 2 |  | Intron4 | G=0.0023  A=0.9977 |
| chr5:50,687,037 | unknown | 0 | 2 |  | Intron4 |  |
| chr5:50,687,087 | rs182071569 | 0 | 1 |  | Intron4 | T=0.0009  G=0.9991 |
| chr5:50,687,096 | unknown | 0 | 1 |  | Intron4 |  |
| chr5:50,687,338 | rs79564128 | 0 | 12 |  | Intron5 | T=0.0197  C=0.9803 |
| chr5:50,687,419 | rs61557039 | 0 | 2 |  | Intron5 | G=0.0083  A=0.9917 |
| chr5:50,689,527 | rs139406174 | 0 | 2 |  | 3’-UTR | A=0.0018  G=0.9982 |
| chr5:50,689,770 | unknown | 0 | 1 |  | 3’-UTR |  |
| chr5:50,689,983 | rs41268421 | 0 | 20 |  | 3’-UTR | T=0.0257  G=0.9743 |
| chr5:50,690,095 | rs1017 | 64 | 158 |  | 3’-UTR | T=0.3669  A=0.6331 |

“Homo” or “Het” columns indicate how many cases of “Homozygous” or “Heterozygous” nucleotide changes compared to reference nucleotides were identified from the 389 cases.

Supplemental Figure 1: Distribution of SNPs identified by Sequencing


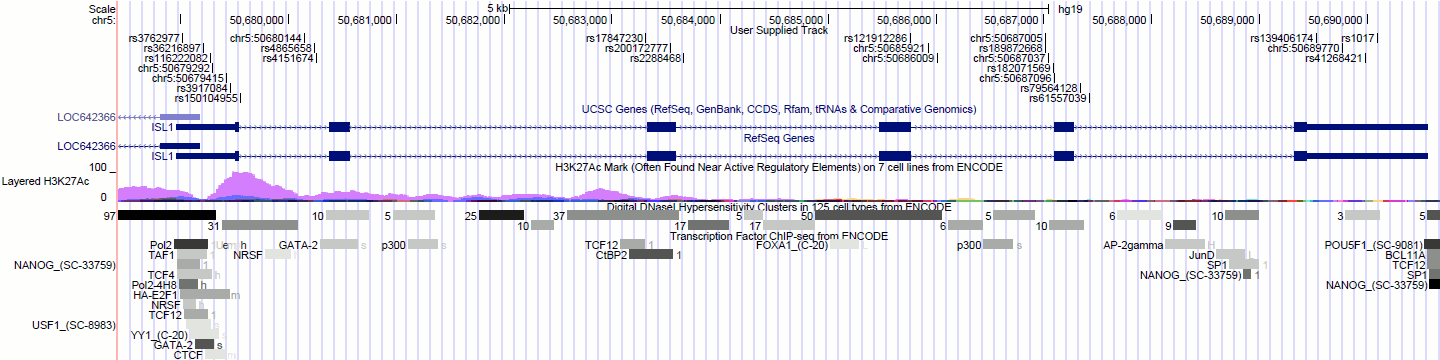


The known and novel variations identified in Supplemental Table 3 are displayed on UCSC Genome Bowser using “custom track” function. The “Integrated Regulation from ENCODE tracks” were activated to determine whether these SNPs are located within the putative regulatory elements.
